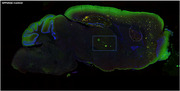# Cerebral Amyloid Angiopathy like pathology accompanied by intense gliosis in T2D‐induced APPxhQC mice

**DOI:** 10.1002/alz.087864

**Published:** 2025-01-03

**Authors:** Joshua Babalola, Joerg Neddens, Gerd Leitinger, Birgit Hutter‐Paier, Gerald Hoefler

**Affiliations:** ^1^ D&F Institut für Pathologie, Medizinische Universität Graz, Graz Austria; ^2^ QPS Austria GmbH, Grambach Austria; ^3^ Division of Cell Biology, Gottfried Schatz Research Center, Medizinische Universität Graz, Graz Austria

## Abstract

**Background:**

Cerebral Amyloid Angiopathy (CAA), characterized by the presence of amyloid β (Aβ) deposits in cerebral blood vessels has been associated with cognitive impairment and Alzheimer’s disease (AD). Vascular risk factors, such as type 2 diabetes (T2D), are known to affect vascular pathology and CAA‐like depositions. Furthermore, Aβ deposition in blood vessels accompanied my inflammation especially gliosis, has been reported in the transgenic AD mouse models 5xFAD and APP_SL_ that express human Aβ. In this study, we therefore investigated the prevalence of CAA‐like pathology in T2D‐induced APPxhQC transgenic mice.

**Method:**

Male transgenic APPxhQC mice, expressing human APP751 with the Swedish and the London mutation and human glutaminyl cyclase (hQC) enzyme, were used. Mice were either fed with high fat (HFD) or control diet and daily treated with streptozocin for 3 days. Brain concentrations of soluble and insoluble Aβ _1‐38_, _1‐40_ and _1‐42_ peptides were determined using an immunosorbent assay. Aβ deposits and gliosis were detected by immunofluorescent labelling.

**Results:**

Exceptionally strong but hollow Aβ deposits in the thalamus that go in medio‐lateral direction were found surrounding deposit free areas. These hollow Aβ deposits were accompanied by intense microglia and astrocytic activation upon HFD‐streptozocin treatment. The location and course of these hollow deposits indicated that they surrounded blood vessels. Additionally, plaque‐associated insoluble Aβ_40_ deposits in T2D‐induced APPxhQC mice were significantly higher compared to the control group.

**Conclusion:**

Presence of Aβ deposits surrounding blood vessels accompanied by intense gliosis seems common in APPxhQC mice and might indicate a CAA‐like pathology specific to this AD model.